# Blockade of mGluR5 in astrocytes derived from human iPSCs modulates astrocytic function and increases phagocytosis

**DOI:** 10.3389/fimmu.2023.1283331

**Published:** 2023-12-11

**Authors:** Izabella B. Q. de Lima, Pablo L. Cardozo, Julia S. Fahel, Juliana P. S. Lacerda, Aline S. Miranda, Antônio L. Teixeira, Fabiola M. Ribeiro

**Affiliations:** ^1^ Department of Biochemistry and Immunology, Institute of Biological Sciences (ICB), Universidade Federal de Minas Gerais, Belo Horizonte, Brazil; ^2^ Department of Morphology, Institute of Biological Sciences (ICB), Universidade Federal de Minas Gerais, Belo Horizonte, Brazil; ^3^ Neuropsychiatry Program, Department of Psychiatry and Behavioral Sciences, University of Texas Health Science Center at Houston, Houston, TX, United States

**Keywords:** mGluR5, astrocyte, hiPSC, phagocytosis, TNFα

## Abstract

TNF-α is essential for induction and maintenance of inflammatory responses and its dysregulation is associated with susceptibility to various pathogens that infect the central nervous system. Activation of both microglia and astrocytes leads to TNF-α production, which in turn triggers further activation of these cells. Astrocytes have been implicated in the pathophysiology of a wide range of neurodegenerative diseases with either harmful or protective roles, as these cells are capable of secreting several inflammatory factors and also promote synapse elimination and remodeling. These responses are possible because they sense their surroundings via several receptors, including the metabotropic glutamate receptor 5 (mGluR5). Under neuroinflammatory conditions, mGluR5 activation in astrocytes can be neuroprotective or have the opposite effect. In the current study, we investigated the role of mGluR5 in hiPSC-derived astrocytes subjected to pro-inflammatory stimulation by recombinant TNF-α (rTNF-α). Our results show that mGluR5 blockade by CTEP decreases the secreted levels of pro-inflammatory cytokines (IL-6 and IL-8) following short rTNF-α stimulation, although this effect subsides with time. Additionally, CTEP enhances synaptoneurosome phagocytosis by astrocytes in both non-stimulated and rTNF-α-stimulated conditions, indicating that mGluR5 blockade alone is enough to drive synaptic material engulfment. Finally, mGluR5 antagonism as well as rTNF-α stimulation augment the expression of the reactivity marker SERPINA3 and reduces the expression of synaptogenic molecules. Altogether, these data suggest a complex role for mGluR5 in human astrocytes, since its blockade may have beneficial and detrimental effects under inflammatory conditions.

## 1 Introduction

Astrocytes are the most abundant glial cells of the mammalian central nervous system (CNS), playing important roles in CNS physiology and being implicated in the pathology of a diverse array of neurological diseases (1, 2). Astroglia is formed by a highly heterogeneous population of astrocytes (3). These cells may assume a reactive phenotype in pathological contexts, releasing inflammatory factors that might impact disease outcomes either in a detrimental or beneficial manner (4, 5). One important role played by glial cells is the phagocytosis of dead cells, synapses and myelin (6, 7). Although aberrant synapse pruning was shown to contribute to dementia (8), elimination of dystrophic synapses and dendrites, as well as phagocytosis of extracellular protein aggregates, such as amyloid-β (Aβ) (9, 10), is essential for proper brain function. Another important function of astrocytes is to promote synapse remodelling by secreting synaptogenic molecules, including brain derived neurotrophic factor (BDNF) (11), glypican-4 (GPC4) (12), hevin (13), and thrombospondins (TSPs) (13, 14).

The metabotropic glutamate receptor type 5 (mGluR5) is a G-protein-coupled receptor (GPCR) whose involvement in neurodegenerative disorders has been widely studied (15). As in neurons, mGluR5 stimulation in astrocytes activates the Gα_q/11_/PLCβ/IP_3_ pathway, increasing intracellular Ca^2+^, which facilitates glutamate release (16–19). Moreover, stimulation of this receptor activates non-canonical cell signalling pathways, including mitogen-activated protein kinase (MAPK) and phospholipase D (PLD) (20, 21). Although mGluR5 expression in astrocytes declines postnatally (22, 23), previous works have shown an upregulation of this receptor in astrocytes in neurological diseases that most commonly affect adults or the elderly, such as amyotrophic lateral sclerosis (ALS) (24), multiple sclerosis (MS) (25), and Alzheimer’s disease (AD) (26), suggesting a relevant role of mGluR5 in gliopathology. In microglia, the activation of mGluR5 has an anti-inflammatory effect, decreasing microglial expression of tumor necrosis factor-α (TNF-α) and production of reactive oxygen species (27, 28). However, the role of mGluR5 in astrocytes remains controversial, as some studies indicate that activation of astrocytic mGluR5 following injury is protective for neighbouring cells as it triggers the release of growth factors and synaptogenic molecules (29, 30), whereas others have shown that mGluR5 activation is harmful as it increases the production of inflammatory mediators (31, 32). For instance, the inhibition of mGluR5 by MPEP prevents methamphetamine-induced increase in IL-6 and IL-8 in a human astroglia cell line (32). Conversely, inhibition of mGluR5 in murine astrocytes can reduce the expression of IL-1β and MCP-1 (33). Furthermore, it has been shown that the activation of mGluR5 by DHPG has no effect on the IL-1β-induced expression of IL-6 in human astrocytes (34). Despite all these studies investigating the role of astrocytic mGluR5 in neurological disorders, it has been challenging to successfully replicate these findings in clinical trials. This discrepant results can be partially explained by the molecular differences between human and murine glial cells (35, 36). For instance, it has been pointed that these cells display species-specific gene expression profiles upon poly-I:C or TNF-α stimulation, with human astrocytes showing stronger immune response compared to their murine counterparts (35). Thus, more studies are needed to investigate how mGluR5 can impact the activation of human astrocytes and the production of inflammatory and synaptogenic mediators.

Astrocytes derived from human induced pluripotent stem cells (hiPSCs) are widely used to study cell physiology, as well as an *in vitro* model of neurological diseases (37–40). In this study we used cultured hiPSC-derived astrocytes to investigate the influence of mGluR5 on the astrocytic response to inflammatory stimulation with recombinant TNF-α (rTNF-α). TNF-α is essential for induction and maintenance of inflammatory responses and its dysregulation is associated with susceptibility to various CNS pathogens (41, 42). Moreover, reactive astrocytes lead to TNF-α production, which in turn triggers further activation of these cells. To modulate mGluR5 activity, we employed the mGluR5 negative allosteric modulator (NAM) CTEP, which is a potent and highly specific drug that acts as an inverse agonist of mGluR5, effectively blocking its constitutive activity (43). We show that, in the presence of rTNF-α, mGluR5 inhibition decreases the secreted levels of IL-6 and IL-8 and increases the expression of the reactive astrocyte marker *SERPINA3*. Since reactive astrogliosis can display different functional profiles (44) and given its role in synaptic turnover, a process altered in many neurological diseases (45), we also assessed the rate of phagocytosis of synaptoneurosomes by astrocytes and the expression of synaptogenic proteins. We show that CTEP enhanced phagocytosis regardless of rTNF-α, while both CTEP and rTNF-α downregulated the expression of synaptogenic molecules. Thereby, the current results reveal the multifaceted role of mGluR5 in astrocyte reactivity and function, highlighting its potential for both detrimental and beneficial effects depending on the specific context.

## 2 Materials and methods

### 2.1 Human-induced pluripotent stem cell line and cell culture maintenance

The 7889SA cell line was obtained from the New York Stem Cell Foundation (NYSCF ID CO0002-01-SV-003) and described previously (46). hiPSCs were maintained on 6-well plates coated with Geltrex (Gibco, cat no. A1413302) in StemFlex Medium (Gibco, cat no. A3349301). Upon reaching 80-90% confluence, cells were incubated for 3-4 min in 500 µM EDTA (Sigma, cat no. EDS) in PBS, dissociated in clumps and seeded into new 6-well Geltrex-coated plates for expansion. Media was changed every 48h.

### 2.2 Human astrocytes differentiation

Astrocytes were differentiated from hiPSCs as previously described (38). hiPSCs were dissociated into single cells using StemPro Accutase (Gibco, cat no. A1110501) and seeded into 6-well Geltrex-coated plates at 3x10^4^ cells/cm^2^ in StemFlex Medium supplemented with 10 µM Rho-associated protein kinase (ROCK) inhibitor Thiazovivin (Sigma Aldrich, cat no. SML1045). In order to start differentiation (day 0), medium was changed to PSC Neural Induction Medium containing Neurobasal Medium (Gibco, cat no. 21103-049), Neural Induction Supplement (Gibco, cat no. A1647701) and 1% penicillin-streptomycin solution (Gibco, cat no. 15410-122). Media was changed every 48 h. On day 7, NSCs were dissociated with Accutase and plated at 1×10^5^ cells/cm^2^ in 60 mm Geltrex-coated dishes in NSC Expansion Medium containing 50% Advanced DMEM/F12 (Gibco, cat no. 12634010), 50% Neurobasal Medium, Neural Induction Supplement and 1% penicillin-streptomycin solution supplemented with 10 µM ROCK inhibitor Thiazovivin. Media changes were performed every 48h. When NSCs reached 90% confluence, cells were dissociated with Accutase and seeded at 5 × 10^4^ cells/cm^2^ in 25 cm^2^ Geltrex-coated culture flasks in Astrocyte Induction Medium containing DMEM/F12 Medium (Gibco, cat no.12400024), N2 supplement (Gibco, cat no. 17502048), 1% fetal bovine serum (FBS) (Gibco, cat no. 12657-029) and 1% antibiotic-antimycotic solution (Gibco, cat no.15240112). Media was changed every 48 h for 21 days. During this period, upon reaching full confluence, cells were expanded at a ratio of 1:3 using Accutase to 75 cm^2^ Geltrex-coated culture flasks. By the end of differentiation, media was switched to Astrocyte Maturation Medium (DMEM/F12, 10% FBS and 1% antibiotic-antimycotic solution) for an additional period of at least 5 weeks. During this period, media was changed twice a week and when reaching full confluence, cells were dissociated using Trypsin/EDTA 0,125% (Gibco, cat no. 25200072) and expanded to 175 cm^2^ culture flasks at a ratio of 1:2 (without Geltrex coating). Cells were maintained under standard culture conditions (95% relative humidity and 5% CO2 at 37°C) and tested routinely for *Mycoplasma* contamination, as previously described (47).

### 2.3 Drug treatment

After nine weeks of maturation, upon reaching 100% confluence in 175 cm^2^ culture flasks, hiPSC-derived astrocytes were dissociated with Trypsin/EDTA 0.125% (Gibco, cat no. 25200072) and seeded at 1x10^4^ cells/cm^2^ in 6-well plates. After 5 days in culture, cells were washed three times with PBS, DMEM/F12 media was replenished, and astrocytes were serum starved for 24 h. Then, cells were treated with either 10 µM CTEP (Axon Medchem, cat no. Axon 1972) or vehicle (DMSO; Sigma-Aldrich, cat no. 41639) and subsequently stimulated with 10 ng/mL rTNF-α (BioLegend, cat no. 717904) for either 4h or 24 h. In the case of lipopolysaccharide (LPS) treatment, astrocytes were treated with either 0.1 or 1 µg/mL LPS (Sigma, cat no. L6529) for 24 h. Drugs were kept in the media throughout the whole experiment. After treatment, the supernatant was flash frozen and stored at -80°C until further use, and cells were collected with 1 mL of TRIzol^™^ reagent (Invitrogen, cat no. 15596018), transferred to 1.5 mL microcentrifuge tubes and frozen at -80°C.

### 2.4 Quantitative RT-PCR

Total RNA was isolated using TRIzol™ reagent as per manufacturer’s instructions and resuspended in 12 μL of nuclease-free water. RNA concentration and quality was analyzed by spectrophotometer (Multiskan^®^ GO, Thermo Scientific). cDNAs were prepared from 800 ng of total RNA extracted in a 20 µL final reverse transcription reaction. Quantitative RT-PCR (RT-qPCR) was performed with 10x diluted cDNA using Power SYBR^®^ Green PCR Master Mix in the QuantStudio^™^ 7 Flex real-time PCR system platform (Applied Biosystems^®^). RT-qPCR assays were performed to quantify the mRNA levels of the following genes: *C3* (NM_000064.3); *IPO8* (NM_006390.3); *RPLP0* (NM_001002.3); *MERTK* (NM_006343.2); *ITGAV* (NM_001144999.3); *SERPINA3* (NM_001085.5); *VCAM1* (NM_001078.5); *S100A10* (NM_002966.3); *BDNF* (NM_170735.6); *GPC4* (NM_001448.3); *TSP1* (NM_003246.4), *FKBP5* (NM_001145775.3), *SERPING1* (NM_000062.3), *GBP2* (NM_004120.5), *NFATC3* (NM_173163.3), *NFATC4* (NM_001198967.3), and *GRM5* (NM_001384268.1). Primers were designed using the Primer3Plus Program (48). Primer sequences are listed in **Table 1**.

**Table 1:** qPCR primer sequences.

Gene	Forward primer (5’-3’)	Reverse primer (5’-3’)
C3	CTGCCCAGTTTCGAGGTCAT	CGAGCCATCCTCAATCGGAA
IPO8	TCCGAACTATTATCGACAGGACC	GTTCAAAGAGCCGAGCTACAA
RPLP0	TTAAACCCTGCGTGGCAATC	ATCTGCTTGGAGCCCACATT
MERTK	TGGCGTAGAGCTATCACTG	CTGGCGTGAGGAAGGGATAA
ITGAV	AATCTTCCAATTGAGGATATCAC	AAAACAGCCAGTAGCAACAAT
SERPINA3	CCTGAGGCAGAGTTGAGAATGG	TCAAGTGGGCTGTTAGGGTG
VCAM1	CGAACCCAAACAAAGGCAGA	ACAGGATTTTCGGAGCAGGA
S100A10	AACAAAGGAGGACCTGAGAGTAC	CTTTGCCATCTCTACACTGGTCC
BDNF	AGTTGGGAGCCTGAAATAGTGG	AGGATGCTGGTCCAAGTGGT
GPC4	GTCAGCGAACAGTGCAATCAT	ACATTTCCCACCACGTAGTAAC
TSP1	GCCAACAAACAGGTGTGCAA	GCAGATGATGCCATTGCCAG
IL-6	AGAGGCACTGGCAGAAAAC	TGCAGGAACTGGATCAGGAC
IL-8	GAGAGTGATTGAGAGTGGAC	GAATTCTCAGCCCTCTTCAAA
GBP2	AATTAGGGGCCCAGTTGGAAG	AAGAGACGGTAACCTCCTGGT
NFATC3	GCGGCCTGCAGATCTTGAGC	TGATGTGGTAAGCAAAGTGGTGTGGT
NFATC4	CCCCGAGTACAGCAACAAGA	CCTCTTTGCAGATCACAGGC
GRM5	ATGCCGGGTGACATCATTATT	TGAATGCCATACTGTTCACGG

Previous verification of undesired secondary formations or dimers between primers were performed using “OligoAnalyser 3.1” tool (Integrated DNA Technologies^©^), available at https://www.idtdna.com/calc/analyzer. All primers used in this work were validated by serial dilution assay and the reaction efficiency was calculated, comprising 90-110% (data not shown). Changes in gene expression were calculated by the 2^−ΔCt^ method, using the average of the housekeeping genes *IPO8* and *RPLP0* for normalization.

### 2.5 Immunofluorescence staining

After 5 weeks of maturation, hiPSC-derived astrocytes were plated onto acid-etched clean glass coverslips coated with 50 µg/mL poly-D-lysine (Sigma, cat no. P6407) in 24-well plates at 1x10^3^ cells/cm^2^ in Astrocytes Maturation Media. After 5 days in culture, cells were fixed for 15 min in 4% paraformaldehyde (PFA) (Sigma, cat no. 158127) diluted in PBS. Samples were permeabilized with 0.3% Triton X-100 (Labsynth, cat no. T2502) diluted in PBS (PBST) for 10 min and blocked with 2% Bovine Serum Albumin (BSA) (Sigma, cat no. A7906) diluted in PBST (blocking solution) for 1 h at room temperature. Then, cells were washed three times with PBS and incubated overnight at 4°C with the following primary antibodies diluted in blocking solution: anti-mGluR5 (1:100, Millipore, cat no. AB5675), anti-S100β (1:200, Abcam, cat no. ab52642) and anti-GFAP (1:200, Cell Signaling, cat no. 12389). After incubation, cells were washed three times with PBS and incubated for 1 h at room temperature with the following secondary antibody and staining reagents diluted in blocking solution: anti-Rabbit IgG Alexa Fluor 488 (1:400, Invitrogen, cat no. A-11008), Hoechst (1:500, Invitrogen, cat no. H3570) and Alexa Fluor 633 Phalloidin (1:1000, Invitrogen, cat no. A22284). Coverslips were washed three times as mentioned above and mounted on clean glass slides with DAKO Mounting Medium (Agilent Technologies, cat no. S302380-2). Astrocytes were imaged using a Nikon A1 Laser Confocal Microscope (CGB, UFMG, Brazil).

### 2.6 Synaptoneurosomes isolation and staining

Housing and all methods and experimentations were carried out in compliance with the ARRIVE guidelines (49) and according to the guidelines of the Brazilian National Council of Control of Animal Experimentation (CONCEA) and approved by the Ethics Committee on Animal Use (CEUA) of Federal University of Minas Gerais, under the protocol number CEUA #120/2017. A 12-month-old male wild-type C57BL/6 mouse was obtained from UFMG Central Animal Facility. Mouse brain was dissected, weighed, and cut into sections of approximately 100 mg that were individually transferred to an ice-cold sterile Dounce homogenizer with 1 mL of synaptoneurosomes isolation buffer (SIB) (10 mM HEPES, 1 mM EDTA, 2 mM EGTA, 0.5 mM DTT and protease inhibitors, pH 7.0; sterile-filtered) (50). Tissue was broken with 10 slow strokes, homogenates were transferred to 1.5 mL microcentrifuge conical tubes and centrifuged at 1200 g for 10 min at 4°C. The supernatant was transferred to new 1.5 mL microcentrifuge tubes and centrifuged at 15000 g for 20 min at 4°C. The pelleted debris fraction and a small aliquot of the supernatant (synaptoneurosomal homogenate) were collected in SIB and stored at -80°C for later validation. The supernatant (cytosolic fraction) was collected into new 1.5 mL microcentrifuge tubes and frozen at -80°C and the pelleted synaptoneurosomes resuspended in SIB + 5% DMSO (Sigma, cat no. D8418), aliquoted and frozen at -80°C until later use.

On the day of synaptoneurosomal engulfment assay, synaptoneurosome aliquots were thawed and their protein concentration determined via the Bradford Protein Assay (Bio-rad, cat no. 5000205). Synaptoneuromes were spun down by centrifugation at 15000 g for 20 min at 4°C, supernatant was discarded, and the pellet resuspended in 2 µM Vybrant CM-Dil solution (Invitrogen, cat no. V22888) diluted in room temperature sterile PBS. Staining was performed according to manufacturer instructions. CM-Dil-labeled synaptoneurosomes were spun down as described above, washed twice with ice-cold PBS to remove fluorescent dye excess and resuspended in ice-cold PBS adjusting its concentration to 0.25 µg/µL. Synaptoneurosomes were kept on ice and protected from light until its use in the aforementioned assay. All procedures were carried out under sterile conditions.

### 2.7 Synaptoneurosome engulfment assay and analysis

hiPSC-derived astrocytes were plated onto acid-etched clean glass coverslips coated with 50 µg/mL poly-D-lysine in 24-well plates at 5.5x10^3^ cells/cm^2^ in Astrocytes Maturation Media. After 5 days in culture, cells were washed three times with PBS and stained with 10 µM CellTracker Blue CMF_2_HC Dye (Invitrogen, cat no. C12881) diluted in warm DMEM/F12 without FBS and incubated for 45 min in the cell culture incubator. The staining solution was removed, fresh DMEM/F12 media was replenished, and astrocytes serum starved for 22 h. On the following day, cells were pre-treated for 1 h with either 10 µM CTEP or vehicle (DMSO) and subsequently stimulated with 10 ng/mL rTNF-α for an extra hour. Then, 1.875 µg (7.5 µL) of CM-Dil-labeled synaptoneurosomes were added into each well and incubated for 24 h. After incubation, cells were washed twice with room temperature PBS to remove non-engulfed material and fixed for 15 min with 4% PFA solution diluted in PBS. Coverslips were washed three times as mentioned above and mounted on clean glass slides with DAKO Mounting Medium. Images were acquired using a Nikon A1 Laser Confocal Microscope. All assays were carried out in duplicates and twice independently.

Images were analyzed using FIJI (v. 1.53t) and CellProfiler (v. 4.2.5) software programs. Briefly, CellTracker-labeled astrocytes and CM-Dil-labeled synaptoneurosomes corresponding channels were split and images pre-processed using File S1 and File S2 macros in FIJI, respectively, saved and exported in.*tif* format. Pre-processed.*tif* files were imported to CellProfiler and analyzed using the File S3 pipeline. Phagocytic Index (PI) was calculated for each cell using the formula below:


$$PI=\frac{Engulfed\;synaptoneurosomes\;area\;(\mu m^{2})}{Total\;cell\;area\;(\mu m^{2})}$$

Orthogonal projections and 3D rendering (**Supplementary Figure S5** and **Movie S1**) of z-stacks were generated using FIJI and FluoRender (v. 2.29.2), respectively.

### 2.8 Immunoblotting

Protein concentration of synaptoneurosomal preparation fractions (pelleted debris, synaptoneurosomal homogenate, cytosolic fraction and isolated synaptoneurosomes) was measured using the Bradford Protein Assay. Twenty-five µg of each fraction was diluted in Laemmli Sample Buffer, boiled at 95°C for 5 min and resolved in 10% SDS-PAGE. Proteins were transferred onto a 0.45 µm nitrocellulose membrane (Bio-Rad, cat no. 1620115), blocked with 5% BSA and 0.1% Tween-20 (Labsynth, cat no. T1028) diluted in TBS (TBST) for 1 h at room temperature, followed by overnight incubation at 4°C with the following primary antibodies in 3% BSA solution diluted in TBST: anti-syntaxin1 (1:200, Santa Cruz, cat no. sc-12736), anti-Homer (1:500, Santa Cruz, sc-8921) and anti-vinculin (1:10000, Abcam, ab129002). After incubation, primary antibodies were removed, membranes washed three times with TBST and incubated for 1 h at room temperature with secondary antibodies in 3% free-fat milk diluted in TBST: HRP-conjugated anti-mouse IgG (1:2500, Millipore, cat no. AP308P), HRP-conjugated anti-rabbit IgG (1:2500, Bio-Rad, cat no. 1706515) and HRP-conjugated anti-goat IgG (1:2500, Santa Cruz, sc-2354). Afterwards, membranes were washed three times as already described and incubated for 5 min with ECL Prime Western Blot Detection Reagent (Cytiva, cat no. RPN2232) for chemiluminescence detection using the ImageQuant LAS 4000 (GE Healthcare) platform.

### 2.9 Cytokine quantification

Cytokines were quantified in cell culture supernatants by flow cytometry using the BD Cytometric Bead Array Human Inflammatory Cytokines Kit (Becton, Dickinson and company - BD Biosciences, cat no. 551811), according to manufacturer’s instructions. Briefly, equal amounts of each capture bead for human IL-1β, IL-6, IL-8, IL-10, TNF-α and IL12-p70 were mixed into a single tube. Fifty µL of the capture beads mix were added to assay tubes followed by addition of 50 µL of cell culture supernatants or cytokine standard dilutions. Then, 50 µL of PE detection reagent were added and assay tubes were incubated protected from light for 3 hours at room temperature. After incubation, samples were washed with 1 mL of wash buffer and tubes were centrifuged at 200** g** for 5 minutes. Supernatants were aspirated and discarded and the bead pellets were resuspended in 300 µL of wash buffer. Sample acquisition was performed on the FACS Aria Fusion (BD Biosciences). The CBA Analysis Software (BD Biosciences) was used for data analysis based on standard concentration curves and the results were expressed as pg/mL.

### 2.10 Statistical analyses

Statistical analyses and data plots were performed using the GraphPad Prism (v. 8.0.1) software. Two-way ANOVA, followed by Tukey’s multiple comparison tests with confidence level set to 0.95 (α = 0.05) as the lowest accepted limit was carried out for all experiments, unless otherwise stated. For the synaptoneurosomes engulfment assay, the Kolmogorov-Smirnov test was executed, indicating the data followed a lognormal distribution. A generalized linear model fitted in the lognormal distribution, followed by Sidak’s multiple comparison test with a confidence level set to 0.99 (α = 0.01) as the lowest accepted limit was employed. This latter analysis was performed with the STATA (v. 14.0) software.

## 3 Results

### 3.1 mGluR5 blockade modulates rTNF-α-induced proinflammatory response in human astrocytes

**Figure 1:**
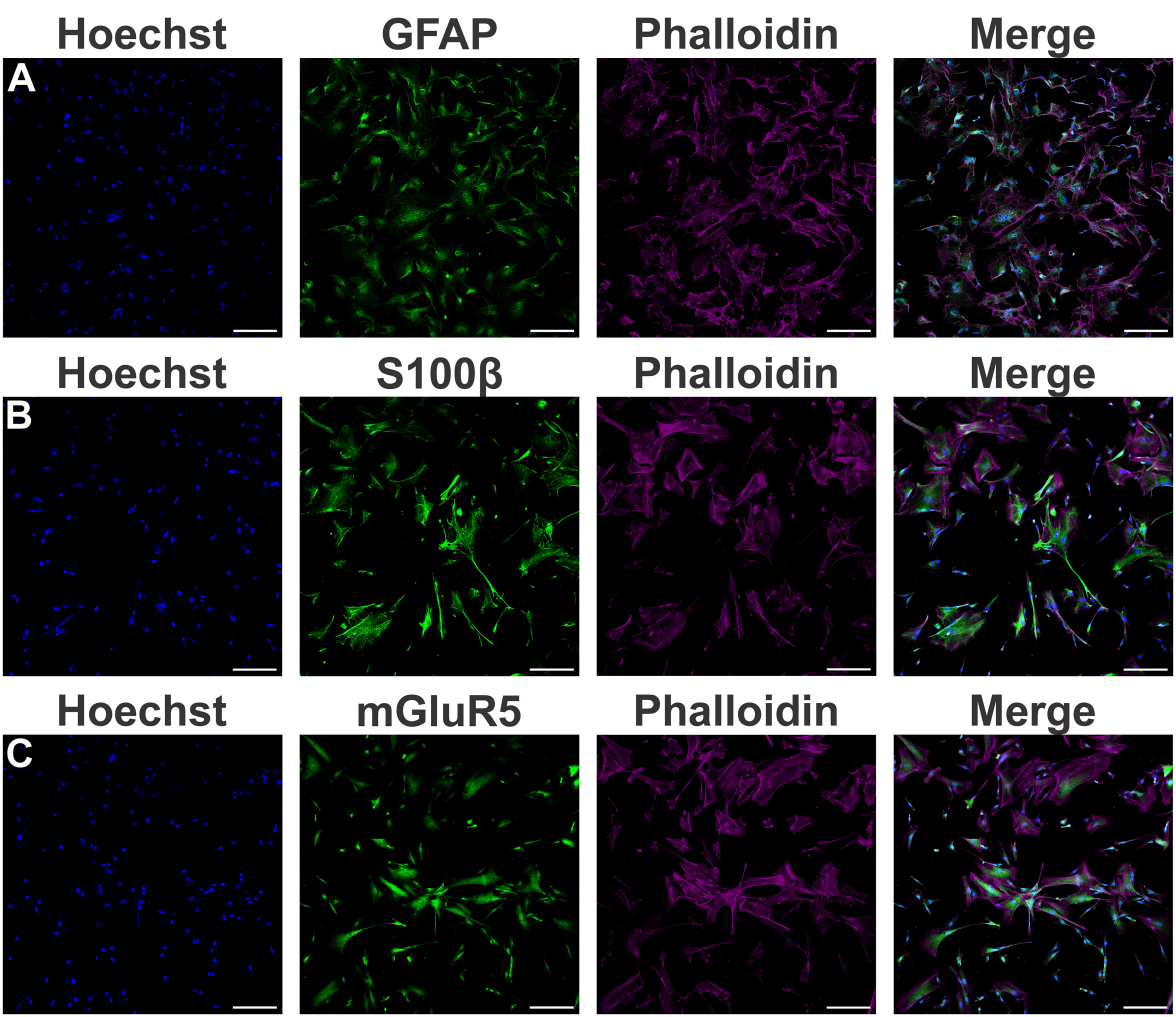
Characterization of astrocytes derived from human induced pluripotent stem cells (hiPSCs). **(A)** Shown are representative laser scanning confocal micrographs from hiPSC-derived astrocytes immunolabeled for phalloidin (magenta), GFAP (green), and Hoechst (blue). **(B)** Shown are representative laser scanning confocal micrographs from hiPSC-derived astrocytes immunolabeled for phalloidin (magenta), S100β (green), and Hoechst (blue). **(C)** Shown are representative laser scanning confocal micrographs from hiPSC-derived astrocytes immunolabeled for phalloidin (magenta), mGluR5 (green), and Hoechst (blue). Scale bar=200 μm.

**Figure 2:**
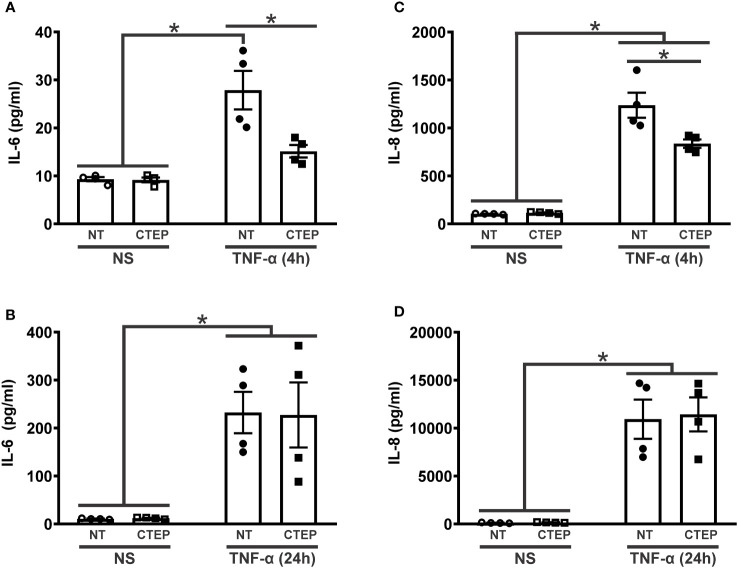
CTEP treatment reduces rTNF-α-induced expression of inflammatory factors. Graphs show protein quantification of IL-6 **(A)** and IL-8 **(C)** in the supernatant of hiPSC-derived astrocytes that were either unstimulated (NS) or stimulated with rTNF-α 10 ng/mL and treated with either vehicle (NT) or CTEP 10 µM for 4 h. Graphs show protein quantification of IL-6 **(B)** and IL-8 **(D)** in the supernatant of hiPSC-derived astrocytes that were either unstimulated (NS) or stimulated with rTNF-α 10 ng/mL and treated with either vehicle (NT) or CTEP 10 µM for 24 h. Protein levels were assessed by CBA, which was performed in duplicates. Data represents the means ± SEM, n=4-6. * (p<0.05) indicates significant differences.

Little is known about mGluR5 function in human cells. In order to investigate the role of mGluR5 in human astrocytes under proinflammatory conditions, astrocytes were differentiated from hiPSCs for four weeks and matured for an additional period of five weeks until they displayed strong expression of the canonical astrocytic markers GFAP (**Figure 1A**) and S100β (**Figure 1B**). In addition, these hiPSC-derived astrocytes also showed mGluR5 mRNA (**Supplementary Figure S1**) and protein (**Figure 1C**) expression. Stimulation of hiPSC-derived astrocytes with LPS 1 µg/mL led to only a marginal increase in IL-6 mRNA levels, whereas IL-8 expression remained unmodified (**Supplementary Figure S2A and S2B**). However, following stimulation with rTNF-α 10 ng/mL, astrocytes increased the mRNA levels of both IL-6 and IL-8 (**Supplementary Figure S2C and S2D**). Thus, we decided to employ rTNF-α in this study to induce an inflammatory response. To further evaluate cell activation, we stimulated astrocytes with rTNF-α 10 ng/mL and measured the production of inflammatory factors in the cell culture supernatant. Upon stimulation with rTNF-α, astrocytes quickly responded by up-regulating IL-6 and IL-8 levels (**Figure 2**), while IL-1β, IL-10 and IL-12p70 secretion was not detected. Both IL-6 and IL-8 secreted protein levels displayed a modest elevation at 4 h (**Figures 2A, C**), followed by a marked increase at 24 h post-stimulation (**Figures 2B, D**).

Blocking mGluR5 activity with CTEP (10 µM) led to a reduction in IL-6 and IL-8 secretion only at 4 h post-stimulation (**Figures 2A, C**), with this effect wearing off at 24h (**Figures 2B, D**). Moreover, treatment with either rTNF-α or CTEP did not alter mGluR5 expression in hiPSC-derived astrocytes (**Supplementary Figure S1**). These data indicate that mGluR5 antagonism can prevent pro-inflammatory cytokine production by astrocytes during a short rTNF-α exposure.

### 3.2 mGluR5 modulates the expression of the reactive astrocyte marker SERPINA3 in human astrocytes

**Figure 3:**
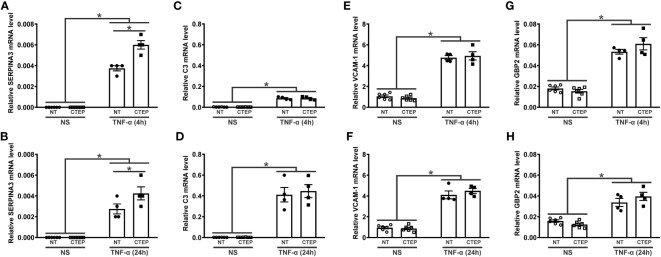
mGluR5 modulates the expression of the reactive astrocyte marker *SERPINA3* without modifying the expression of A1 and A2 markers. Graphs show mRNA levels of *SERPINA3* **(A)**, *C3* **(C)**, *VCAM-1* **(E)**, and *GBP2* **(G)** in hiPSC-derived astrocytes that were either unstimulated (NS) or stimulated with rTNF-α 10 ng/mL and treated with either vehicle (NT) or CTEP 10 µM for 4 h. Graphs show mRNA levels of *SERPINA3* **(B)**, *C3* **(D)**, *VCAM-1* **(F)**, and *GBP2* **(H)** in hiPSC-derived astrocytes that were either unstimulated (NS) or stimulated with rTNF-α 10 ng/mL and treated with either vehicle (NT) or CTEP 10 µM for 24 h. mRNA levels were assessed by quantitative RT-PCR, which was performed in triplicates and normalized to the average of *RPLP0* and *IPO8* mRNA levels. Data represents the means ± SEM, n=4-6. * (p<0.05) indicates significant differences.

It has been proposed that astrocytes can be polarized to either an inflammatory and neurotoxic (A1 astrocytes) or a neuroprotective (A2 astrocytes) phenotype in response to external stimulation (51–53). Accordingly, we analyzed the expression of the general reactive astrocyte markers *SERPINA3*, *NFATC3*, and *NFATC4*; the A1-reactive markers *C3*, vascular cell adhesion molecule 1 (*VCAM-1*), *GBP2*, *FKBP5*, and *SERPING1*; and the A2-reactive marker *S100A10* (40, 51–53) in human astrocytes. rTNF-α stimulation promoted an augmentation in *SERPINA3*, *C3*, *VCAM-1*, and *GPB2* transcript levels at all tested timepoints (**Figure 3**), while the transcriptional levels of *NFATC3*, *NFATC4*, *FKBP5*, *SERPING1*, and *S100A10* remained unchanged relative to non-stimulated astrocytes (**Supplementary Figure S3**). Notably, CTEP treatment led to an even greater up-regulation of *SERPINA3* gene expression in rTNF-α-stimulated astrocytes at all tested timepoints (**Figures 3A, B**). Additionally, CTEP treatment did not modify the expression of *FKBP5*, *SERPING1*, *NFATC3*, *NFATC4*, and *S100A10* (**Supplementary Figure S3**) or induce further alterations in the expression of the reactive astrocyte markers *C3*, *VCAM-1* and *GBP2*, either in the presence or in the absence of rTNF-α stimulation (**Figures 3C–H**). Therefore, mGluR5 blockade promotes a sustained upregulation of the reactive astrocyte marker *SERPINA3* under pro-inflammatory conditions, without changing the expression of A1- and A2-reactive markers.

### 3.3 CTEP treatment leads to enhanced synaptic material engulfment by human astrocytes

It has been shown that astrocyte reactivity affects its capacity to uptake synaptic material (52). Then, we decided to develop an assay to assess synaptic material phagocytosis by the hiPSC-derived astrocytes. First, we isolated synaptoneurosomes from mouse brain and evaluated if this procedure was effective in enriching both pre- and post-synaptic proteins. As seen in **Supplementary Figure S4**, both syntaxin-1 and Homer, respectively, pre- and postsynaptic markers, were enriched in the synaptoneurosomes fraction, while vinculin, a cytoskeleton protein, was mostly confined to the cytosolic compartment. Afterwards, we investigated whether the hiPSC-derived astrocytes could engulf fluorescently-labeled synaptoneurosomes. Human astrocytes were capable to engulf synaptic material, as several red *puncta*, corresponding to fluorescently-labeled synaptoneurosomes, were observed inside the astrocyte cell bodies (**Supplementary Figure S5** and **Movie S1**).

**Figure 4:**
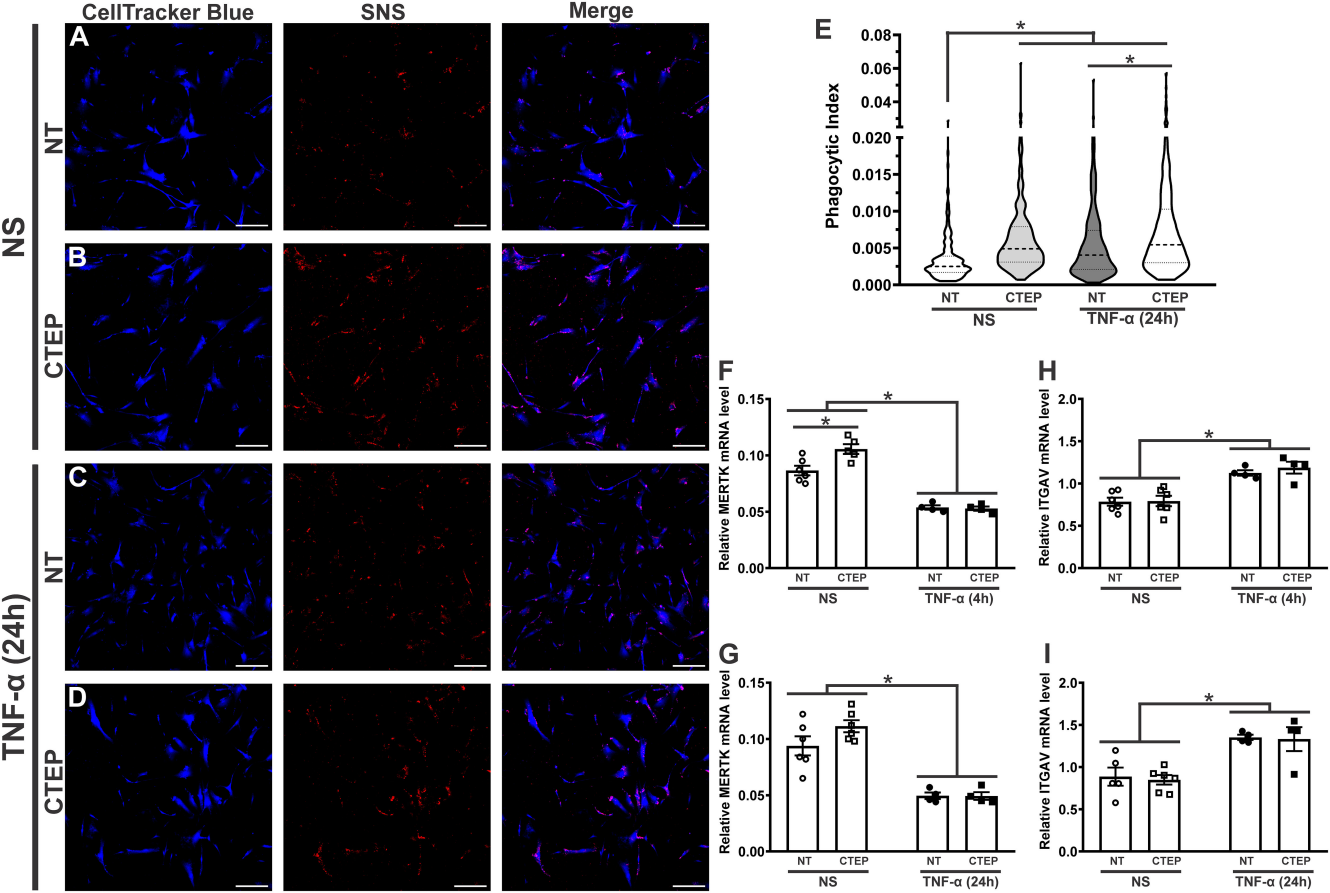
Both CTEP and rTNF-α increase astrocytic phagocytosis. Shown are representative laser scanning confocal micrographs from hiPSC-derived astrocytes labelled with CellTracker blue and synaptoneurosomes (SNS) labelled with Vybrant CM-Dil (red). hiPSC-derived astrocytes were either unstimulated (NS), and treated with either vehicle (NT) **(A)** or CTEP 10 µM **(B)**, or stimulated with rTNF-α 10 ng/mL, and treated with either vehicle (NT) **(C)** or CTEP 10 µM **(D)** for 24 h. Scale bar=200 μm. **(E)** Graph shows phagocytic index of hiPSC-derived astrocytes that were either unstimulated (NS) or stimulated with rTNF-α 10 ng/mL and treated with either vehicle (NT) or CTEP 10 µM for 24 h. Dashed line represents median and dotted lines represent interquartile interval, n=258-414. Graphs show mRNA levels of *MERTK* **(F)** or *ITGAV* **(H)** in hiPSC-derived astrocytes that were either unstimulated (NS) or stimulated with rTNF-α 10 ng/mL and treated with either vehicle (NT) or CTEP 10 µM for either 4 h. Graphs show mRNA levels of *MERTK* **(G)** or *ITGAV* **(I)** in hiPSC-derived astrocytes that were either unstimulated (NS) or stimulated with rTNF-α 10 ng/mL and treated with either vehicle (NT) or CTEP 10 µM for 24 h **(I)**. mRNA levels were assessed by quantitative RT-PCR, which was performed in triplicates and normalized to the average of *RPLP0* and *IPO8* mRNA levels. Data represents the means ± SEM, n=4-6. * (p<0.05) indicates significant differences.

rTNF-α stimulation significantly increased astrocytic phagocytosis compared to non-stimulated cells (**Figures 4A, C, E**). As both rTNF-α and CTEP modified the expression of reactive astrocyte markers, next we analyzed whether mGluR5 blockade influences astrocytic phagocytosis. CTEP treatment led to enhanced synaptoneurosomal phagocytosis in human astrocytes in basal (no rTNF-α) conditions (**Figures 4A, B, E**). Interestingly, CTEP-treated astrocytes stimulated with rTNF-α showed even higher synaptic material engulfment (**Figures 4C–E**). In addition, both non-stimulated and rTNF-α-stimulated astrocytes that were pre-treated with 10 µM CTEP displayed similar synaptoneurosomal phagocytic level (**Figures 4B, D, E**). These data suggest that blocking mGluR5 activity is enough to enhance synaptic material engulfment by human astrocytes, even in the absence of proinflammatory stimulation.

Tyrosine-protein kinase mer (MERTK) and αvβ3/5 integrin, composed by the alpha chain V (ITGAV) and the beta 3/5 components, have been identified as important phagocytic receptors responsible for promoting synapse and myelin engulfment by astrocytes (10, 54, 55). Thus, we analyzed the expression of these receptors in human astrocytes upon rTNF-α stimulation and mGluR5 pharmacological inhibition. CTEP treatment increased *MERTK* gene expression in human astrocytes in basal conditions and this difference was statistically significant at the 4 h timepoint (**Figures 4F, G**). Conversely, rTNF-α stimulation reduced *MERTK* mRNA levels (**Figures 4F, G**), at the same time it enhanced *ITGAV* expression (**Figures 4H, I**) in all treatment groups at both 4 h and 24 h post-stimulation. CTEP treatment did not modify ITGAV expression (**Figures 4H, I**). Therefore, different phagocytic receptors might be involved in TNF-α- and mGluR5-induced phagocytosis by astrocytes.

### 3.4 rTNF-α stimulation and mGluR5 blockade decrease the expression of synaptogenic molecules in human astrocytes

**Figure 5:**
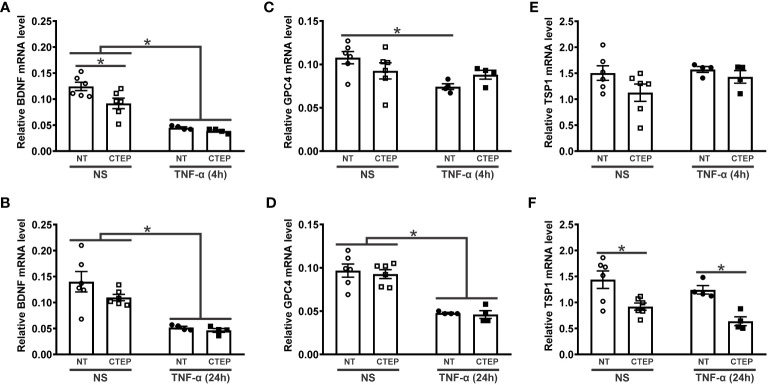
Both CTEP and rTNF-α decrease the expression of synaptogenic molecules. Graphs show mRNA levels of BDNF **(A)**, glypican-4 (GPC4) **(C)**, and trombospoindin-1 (TSP1) **(E)** in hiPSC-derived astrocytes that were either unstimulated (NS) or stimulated with rTNF-α 10 ng/mL and treated with either vehicle (NT) or CTEP 10 µM for 4* h*. Graphs show mRNA levels of BDNF **(B)**, GPC4 **(D)**, and TSP1 **(F)** in hiPSC-derived astrocytes that were either unstimulated (NS) or stimulated with rTNF-α 10 ng/mL and treated with either vehicle (NT) or CTEP 10 µM for 24* h*. mRNA levels were assessed by quantitative RT-PCR, which was performed in triplicates and normalized to the average of RPLP0 and IPO8 mRNA levels. Data represents the means ± SEM, n=4-6. * (p<0.05) indicates significant differences.

The activation of mGluR5 can induce the expression of synaptogenic factors by astrocytes and promote synapse remodeling (30, 56, 57). To investigate whether mGluR5 could modulate the expression of synaptogenic molecules in an inflammatory context, hiPSC-derived astrocytes were subjected to rTNF-α stimulation for 4 h and 24 h followed by the analysis of gene expression of the synaptogenic factors *BDNF*, *GPC4* and *TSP1*. rTNF-α-stimulated astrocytes displayed reduced *BDNF* and *GPC4* mRNA levels at both timepoints (**Figures 5A–D**). *TSP1* expression, on the other hand, was not affected by rTNF-α stimulation (**Figures 5E, F**). Furthermore, CTEP treatment had no impact in *BDNF* and *GPC4* expression when human astrocytes were subjected to rTNF-α stimulation (**Figures 5A–D**). However, CTEP treatment led to a reduction in *BDNF* expression under basal conditions (no rTNF-α) and this difference was significant in the case of the 4 h timepoint (**Figures 5A, B**). In addition, CTEP drove a reduction in *TSP1* gene expression, which was significantly different in the case of the 24 h timepoint (**Figures 5E, F**). Interestingly, this effect was observed regardless of rTNF-α-stimulation, indicating that mGluR5 antagonism alone is responsible for decreasing *TSP1* expression (**Figure 5F**). Altogether, these data indicate that both pro-inflammatory stimulation and mGluR5 negative allosteric modulation can dampen the production of synaptogenic molecules, which may contribute to the synaptic deficits often seen in neurodegenerative disorders (58, 59).

## 4 Discussion

It is well known that mGluR5 is involved in neuroinflammation and neurodegeneration processes and has hence been pointed as a potential pharmacological target for neuroprotection in a variety of neurodegenerative diseases (60). However, the literature has provided conflicting information concerning whether activation of neuronal mGluR5 is either neuroprotective or neurotoxic and whether mGluR5 stimulation in astrocytes would elicit protective or toxic effects on neighbouring cells. While some studies have reported positive effects of astrocytic mGluR5 activation following injury through the actions of growth factors and synaptogenic molecules (29, 30), others have shown that activation of this receptor may elicit harmful effects through the production of cytokines and inflammatory mediators (31, 32). Actually, it is possible that the utility of agonists or antagonists of the receptor may vary based on the underlying condition. For instance, stimulation of mGluR5 in astrocytes can lead to BDNF release, which supports myelin protein synthesis in the cuprizone-induced demyelination mouse model (30). On the other hand, in the case of AD, it was demonstrated that Aβ induces an increase in intracellular Ca^2+^ levels, which can be explained, at least in part, by an increase in mGluR5 expression in astrocytes (26, 61–65). Thus, in this case, mGluR5 antagonism is efficient to reverse Ca^2+^ rise, preventing Aβ-induced astrocytic Ca^2+^ signalling dysfunction (61, 63, 65). Moreover, mGluR5 blockade in cultured astrocytes derived from hSOD1^G93A^, a transgenic mouse model of ALS, was shown to prevent cell death (31). Our results may shed some light on this dichotomy, as we showed that mGluR5 blockade by CTEP decreased the levels of inflammatory factors following short rTNF-α stimulation, with this effect wearing off at 24 h. This is in line with previous reports showing that mGluR5 antagonism prevents the secretion of IL-8 and IL-6 in an astrocytic cell line (32). However, it is surprising that CTEP augmented the gene expression of *SERPINA3*, which is a marker of reactive astrocytes. In addition, CTEP reduced the expression of trophic and synaptogenic molecules, such as *BDNF* and *TSP1*, and did not modify the expression of A1 and A2 reactive astrocyte markers.

Astrocytes play pivotal roles in neuronal survival and synapse remodelling by secreting trophic and synaptogenic factors, including *BDNF* (11), *GPC4* (12), hevin (13), and TSP (14). We showed that rTNF-α stimulation decreased the levels of *BDNF* and *GPC4*, without modifying *TSP1* mRNA levels. A previous study has shown that murine A1 astrocytes, under distinct inflammatory stimuli (IL-1α 3 ng/mL, TNF-α 30 ng/mL, and C1q 400 ng/mL), exhibit increased levels of GPC4 and TSP1/2 (52). While these contrasting results could be explained by differences in inflammatory stimuli, it is also possible that murine and human iPSC-derived astrocytes respond differently to inflammation, highlighting the importance of employing cell models relevant to humans. Interestingly, Although CTEP had an anti-inflammatory effect, mGluR5 blockade did not result in an increase in the expression of the aforementioned synaptogenic factors. In fact, CTEP decreased *BDNF* and *TSP-1* expression levels, which could contribute to further attenuation of synaptogenesis. These results were anticipated, as it has been shown that astrocytic mGluR5 is necessary for glypican-4, *BDNF* and *TSP1* secretion (30, 56, 57). In the case of BDNF, it has been shown that mGluR5 activation enhances the expression of this trophic factor by increasing the phosphorylation of CREB (66–69). In addition, previous data from our laboratory showed that mGluR5 genetic ablation leads to reduced dendritic spine numbers in a Huntington’s disease mouse model (58), while mGluR5 positive allosteric modulation is capable of rescuing this phenotype (69), indicating that mGluR5 stimulation is synaptogenic. Thus, both rTNF-α and mGluR5 blockade may impair synaptogenesis and future studies will be important to determine whether either astrocyte conditioned media or co-culture of these astrocytes with neurons would lead to decreased number of synapses in human cells.

Phagocytosis is important to eliminate dead cells in both physiological and pathological conditions of the CNS. Synapses and myelin are also eliminated by phagocytosis to maintain or refine neural networks during development and adulthood (6, 7). However, aberrant synapse pruning by microglia is suggested to cause undesired synapse loss in AD (8). Although microglia play a major role in phagocytosis, astrocytes also prune synapses by phagocytosis in the developing brain and take up extracellular protein aggregates, such as Aβ (9, 10). In fact, recent findings indicate that hippocampal synapses are preferentially phagocytosed by astrocytes (70). In a mouse model of AD (APP/PS1 mice), dysfunctional synapses are engulfed by Aβ-associated astrocytes, but not microglia (71). However, this beneficial effect is limited, as progressive accumulation of Aβ impairs the phagocytosis of dystrophic synapses by astrocytes and decreases the expression of the phagocytic receptors MERTK and MEGF10 (72, 73). Even though healthy synapses should be preserved, accumulation of faulty synapses can result in an unhealthy synaptic environment, causing alterations in circuit connectivity consequent cognitive impairment and memory loss. The results shown here demonstrate that rTNF-α enhanced phagocytosis by astrocytes and increased the expression of the integrin alpha chain V, encoded by *ITGAV*, which is part of the phagocytic receptor αvβ3/5 integrin (54), whereas *MERTK* expression was decreased. In contrast, murine A1 astrocytes under inflammatory stimuli (IL-1α, TNF-α, and C1q) exhibit suppressed phagocytic activity for synapses and myelin, concomitantly with downregulation of the phagocytic receptors MERTK and MEGF10 (52). These data suggest that the combination of different inflammatory factors might produce contrasting results on the phagocytic activity of astrocytes, adding to the debate of whether this simplified dichotomic classification of astrocytes into A1 and A2 is enough to fully describe the myriad of phenotypes astrocytes can display (44).

Not many studies have addressed the role of mGluR5 on astrocytic phagocytosis. A recent study shows that a silent allosteric modulator (SAM) of mGluR5 prevents synaptic localization of the complement component C1q and synaptic engulfment by astrocytes in an AD mouse model (59). Here we show that CTEP treatment enhanced phagocytosis in the presence and in the absence of rTNF-α. However, although CTEP increased the expression of *MERTK* in the absence of rTNF-α, *MERTK* expression remained reduced in the presence of this inflammatory factor. This increase in *MERTK* expression by CTEP is in line with the enhanced phagocytosis observed in astrocytes treated with CTEP. Thus, CTEP seems to have a prominent effect to induce phagocytosis, regardless of the presence of rTNF-α. Considering that phagocytosis by microglia (74) and astrocytes (72) decline in certain diseases, including AD, CTEP could be an option to compensate impaired phagocytic clearance of Aβ and dystrophic synapses in AD and in other brain disorders caused by protein aggregates.

In conclusion, mGluR5 blockade by CTEP attenuates the rTNF-α-induced secretion of inflammatory factors, including IL-6 and IL-8 in the short-term, although this effect subsides with time. At the same time, CTEP treatment did not modify either A1 or A2 astrocytic markers, while rTNF-α led to an increase in the A1 markers, *C3*, *VCAM-1* and *GBP2*. *SERPINA3* expression and astrocyte phagocytosis were enhanced by both CTEP and rTNF-α, whereas the expression of synaptogenic factors were decreased. Thus, CTEP treatment could be an option when augmented phagocytosis is desired, although it might lead to increased synaptic pruning and diminished synaptogenesis. These data illustrate the complexity of mGluR5 pharmacology and show that the simplified classification of reactive astrocytes into A1 and A2 falls short of capturing their phenotypic diversity.

## Data availability statement

The original contributions presented in the study are included in the article/**Supplementary Material**. Further inquiries can be directed to the corresponding author.

## Ethics statement

Ethical approval was not required for the studies on humans in accordance with the local legislation and institutional requirements because only commercially available established cell lines were used. The animal study was approved by Ethics Committee on Animal Use of the Federal University of Minas Gerais (CEUA #120/2017). The study was conducted in accordance with the local legislation and institutional requirements.

## Author contributions

IL: Formal analysis, Writing – original draft, Writing – review & editing, Investigation, Methodology. PC: Formal analysis, Investigation, Methodology, Writing – original draft, Writing – review & editing. JF: Formal analysis, Investigation, Methodology, Writing – original draft, Writing – review & editing. JL: Investigation, Methodology, Writing – review & editing. AM: Writing – review & editing, Investigation, Data curation, Conceptualization, Resources, Supervision. AT: Writing – review & editing, Investigation, Data curation, Conceptualization, Supervision. FR: Writing – review & editing, Conceptualization, Data curation, Formal analysis, Funding acquisition, Project administration, Resources, Supervision, Validation, Visualization, Writing – original draft.

## References

[1] Lee HG Wheeler MA Quintana FJ. Function and therapeutic value of astrocytes in neurological diseases. Nat Rev Drug Discovery (2022) 21:339–58. doi: 10.1038/s41573-022-00390-x PMC9081171 35173313

[2] de Lima IBQ Ribeiro FM. The implication of glial metabotropic glutamate receptors in alzheimer's disease. Curr Neuropharmacol (2023) 21:164–82. doi: 10.2174/1570159X20666211223140303 PMC10190153 34951388

[3] Verkhratsky A Nedergaard M. Physiology of astroglia. Physiol Rev (2018) 98:239–389. doi: 10.1152/physrev.00042.2016 29351512 PMC6050349

[4] Moulson AJ Squair JW Franklin RJM Tetzlaff W Assinck P. Diversity of reactive astrogliosis in CNS pathology: heterogeneity or plasticity? Front Cell Neurosci (2021) 15:703810. doi: 10.3389/fncel.2021.703810 34381334 PMC8349991

[5] Verkhratsky A Li B Scuderi C Parpura V. Principles of astrogliopathology. Adv Neurobiol (2021) 26:55–73. doi: 10.1007/978-3-030-77375-5_3 34888830 PMC8999877

[6] Trachtenberg JT Chen BE Knott GW Feng G Sanes JR Welker E. Long-term in *vivo* imaging of experience-dependent synaptic plasticity in adult cortex. Nature (2002) 420:788–94. doi: 10.1038/nature01273 12490942

[7] Paolicelli RC Bolasco G Pagani F Maggi L Scianni M Panzanelli P. Synaptic pruning by microglia is necessary for normal brain development. Science (2011) 333:1456–8. doi: 10.1126/science.1202529 21778362

[8] Hong S Beja-Glasser VF Nfonoyim BM Frouin A Li S Ramakrishnan S. Complement and microglia mediate early synapse loss in Alzheimer mouse models. Science (2016) 352:712–6. doi: 10.1126/science.aad8373 PMC5094372 27033548

[9] Koistinaho M Lin S Wu X Esterman M Koger D Hanson J. Apolipoprotein E promotes astrocyte colocalization and degradation of deposited amyloid-beta peptides. Nat Med (2004) 10:719–26. doi: 10.1038/nm1058 15195085

[10] Chung WS Clarke LE Wang GX Stafford BK Sher A Chakraborty C. Astrocytes mediate synapse elimination through MEGF10 and MERTK pathways. Nature (2013) 504:394–400. doi: 10.1038/nature12776 24270812 PMC3969024

[11] Gomez-Casati ME Murtie JC Rio C Stankovic K Liberman MC Corfas G. Nonneuronal cells regulate synapse formation in the vestibular sensory epithelium *via* erbB-dependent BDNF expression. Proc Natl Acad Sci U.S.A. (2010) 107:17005–10. doi: 10.1073/pnas.1008938107 PMC2947909 20837532

[12] Allen NJ Bennett ML Foo LC Wang GX Chakraborty C Smith SJ. Astrocyte glypicans 4 and 6 promote formation of excitatory synapses *via* GluA1 AMPA receptors. Nature (2012) 486:410–4. doi: 10.1038/nature11059 PMC3383085 22722203

[13] Kucukdereli H Allen NJ Lee AT Feng A Ozlu MI Conatser LM. Control of excitatory CNS synaptogenesis by astrocyte-secreted proteins Hevin and SPARC. Proc Natl Acad Sci U.S.A. (2011) 108:E440–449. doi: 10.1073/pnas.1104977108 PMC3156217 21788491

[14] Christopherson KS Ullian EM Stokes CC Mullowney CE Hell JW Agah A. Thrombospondins are astrocyte-secreted proteins that promote CNS synaptogenesis. Cell (2005) 120:421–33. doi: 10.1016/j.cell.2004.12.020 15707899

[15] Ribeiro FM Vieira LB Pires RG Olmo RP Ferguson SS. Metabotropic glutamate receptors and neurodegenerative diseases. Pharmacol Res (2017) 115:179–91. doi: 10.1016/j.phrs.2016.11.013 27872019

[16] Miller S Romano C Cotman CW. Growth factor upregulation of a phosphoinositide-coupled metabotropic glutamate receptor in cortical astrocytes. J Neurosci (1995) 15:6103–9. doi: 10.1523/JNEUROSCI.15-09-06103.1995 PMC6577656 7666194

[17] Pasti L Volterra A Pozzan T Carmignoto G. Intracellular calcium oscillations in astrocytes: a highly plastic, bidirectional form of communication between neurons and astrocytes in situ. J Neurosci (1997) 17:7817–30. doi: 10.1523/JNEUROSCI.17-20-07817.1997 PMC6793927 9315902

[18] Biber K Laurie DJ Berthele A Sommer B Tolle TR Gebicke-Harter PJ. Expression and signaling of group I metabotropic glutamate receptors in astrocytes and microglia. J Neurochem (1999) 72:1671–80. doi: 10.1046/j.1471-4159.1999.721671.x 10098876

[19] Paquet M Ribeiro FM Guadagno J Esseltine JL Ferguson SS Cregan SP. Role of metabotropic glutamate receptor 5 signaling and homer in oxygen glucose deprivation-mediated astrocyte apoptosis. Mol Brain (2013) 6:9. doi: 10.1186/1756-6606-6-9 23406666 PMC3598502

[20] Peavy RD Conn PJ. Phosphorylation of mitogen-activated protein kinase in cultured rat cortical glia by stimulation of metabotropic glutamate receptors. J Neurochem (1998) 71:603–12. doi: 10.1046/j.1471-4159.1998.71020603.x 9681450

[21] Servitja JM Masgrau R Sarri E Picatoste F. Group I metabotropic glutamate receptors mediate phospholipase D stimulation in rat cultured astrocytes. J Neurochem (1999) 72:1441–7. doi: 10.1046/j.1471-4159.1999.721441.x 10098847

[22] Cai Z Schools GP Kimelberg HK. Metabotropic glutamate receptors in acutely isolated hippocampal astrocytes: developmental changes of mGluR5 mRNA and functional expression. Glia (2000) 29:70–80. doi: 10.1002/(SICI)1098-1136(20000101)29:1<70::AID-GLIA7>3.0.CO;2-V 10594924

[23] Sun W McConnell E Pare JF Xu Q Chen M Peng W. Glutamate-dependent neuroglial calcium signaling differs between young and adult brain. Science (2013) 339:197–200. doi: 10.1126/science.1226740 23307741 PMC3569008

[24] Aronica E Catania MV Geurts J Yankaya B Troost D. Immunohistochemical localization of group I and II metabotropic glutamate receptors in control and amyotrophic lateral sclerosis human spinal cord: upregulation in reactive astrocytes. Neuroscience (2001) 105:509–20. doi: 10.1016/S0306-4522(01)00181-6 11672616

[25] Geurts JJ Wolswijk G Bo L van der Valk P Polman CH Troost D. Altered expression patterns of group I and II metabotropic glutamate receptors in multiple sclerosis. Brain (2003) 126:1755–66. doi: 10.1093/brain/awg179 12805104

[26] Shrivastava AN Kowalewski JM Renner M Bousset L Koulakoff A Melki R. beta-amyloid and ATP-induced diffusional trapping of astrocyte and neuronal metabotropic glutamate type-5 receptors. Glia (2013) 61:1673–86. doi: 10.1002/glia.22548 23922225

[27] Byrnes KR Stoica B Loane DJ Riccio A Davis MI Faden AI. Metabotropic glutamate receptor 5 activation inhibits microglial associated inflammation and neurotoxicity. Glia (2009) 57:550–60. doi: 10.1002/glia.20783 PMC2644739 18816644

[28] Loane DJ Stoica BA Pajoohesh-Ganji A Byrnes KR Faden AI. Activation of metabotropic glutamate receptor 5 modulates microglial reactivity and neurotoxicity by inhibiting NADPH oxidase. J Biol Chem (2009) 284:15629–39. doi: 10.1074/jbc.M806139200 PMC2708859 19364772

[29] Fulmer CG VonDran MW Stillman AA Huang Y Hempstead BL Dreyfus CF. Astrocyte-derived BDNF supports myelin protein synthesis after cuprizone-induced demyelination. J Neurosci (2014) 34:8186–96. doi: 10.1523/JNEUROSCI.4267-13.2014 PMC4051974 24920623

[30] Saitta KS Lercher LD Sainato DM Patel A Huang Y McAuliffe G. CHPG enhances BDNF and myelination in cuprizone-treated mice through astrocytic metabotropic glutamate receptor 5. Glia (2021) 69:1950–65. doi: 10.1002/glia.24003 PMC9847144 33811383

[31] Rossi D Brambilla L Valori CF Roncoroni C Crugnola A Yokota T. Focal degeneration of astrocytes in amyotrophic lateral sclerosis. Cell Death Differ (2008) 15:1691–700. doi: 10.1038/cdd.2008.99 18617894

[32] Shah A Silverstein PS Singh DP Kumar A. Involvement of metabotropic glutamate receptor 5, AKT/PI3K signaling and NF-kappaB pathway in methamphetamine-mediated increase in IL-6 and IL-8 expression in astrocytes. J Neuroinflamm (2012) 9:52. doi: 10.1186/1742-2094-9-52 PMC3338363 22420994

[33] Kim H Woo JH Lee JH Joe EH Jou I. 22(R)-hydroxycholesterol induces HuR-dependent MAP kinase phosphatase-1 expression *via* mGluR5-mediated Ca(2+)/PKCalpha signaling. Biochim Biophys Acta (2016) 1859:1056–70. doi: 10.1016/j.bbagrm.2016.05.008 27206966

[34] Aronica E Gorter JA Rozemuller AJ Yankaya B Troost D. Activation of metabotropic glutamate receptor 3 enhances interleukin (IL)-1beta-stimulated release of IL-6 in cultured human astrocytes. Neuroscience (2005) 130:927–33. doi: 10.1016/j.neuroscience.2004.10.024 15652990

[35] Li J Pan L Pembroke WG Rexach JE Godoy MI Condro MC. Conservation and divergence of vulnerability and responses to stressors between human and mouse astrocytes. Nat Commun (2021) 12:3958. doi: 10.1038/s41467-021-24232-3 34172753 PMC8233314

[36] Degl'Innocenti E Dell'Anno MT. Human and mouse cortical astrocytes: a comparative view from development to morphological and functional characterization. Front Neuroanat (2023) 17:1130729. doi: 10.3389/fnana.2023.1130729 37139179 PMC10150887

[37] Kondo T Asai M Tsukita K Kutoku Y Ohsawa Y Sunada Y. Modeling Alzheimer's disease with iPSCs reveals stress phenotypes associated with intracellular Abeta and differential drug responsiveness. Cell Stem Cell (2013) 12:487–96. doi: 10.1016/j.stem.2013.01.009 23434393

[38] Trindade P Loiola EC Gasparotto J Ribeiro CT Cardozo PL Devalle S. Short and long TNF-alpha exposure recapitulates canonical astrogliosis events in human-induced pluripotent stem cells-derived astrocytes. Glia (2020) 68:1396–409. doi: 10.1002/glia.23786 32003513

[39] Zhao J Fu Y Yamazaki Y Ren Y Davis MD Liu CC. APOE4 exacerbates synapse loss and neurodegeneration in Alzheimer's disease patient iPSC-derived cerebral organoids. Nat Commun (2020) 11:5540. doi: 10.1038/s41467-020-19264-0 33139712 PMC7608683

[40] Leng K Rose IVL Kim H Xia W Romero-Fernandez W Rooney B. CRISPRi screens in human iPSC-derived astrocytes elucidate regulators of distinct inflammatory reactive states. Nat Neurosci (2022) 25:1528–42. doi: 10.1038/s41593-022-01180-9 PMC9633461 36303069

[41] Tsenova L Bergtold A Freedman VH Young RA Kaplan G. Tumor necrosis factor alpha is a determinant of pathogenesis and disease progression in mycobacterial infection in the central nervous system. Proc Natl Acad Sci U.S.A. (1999) 96:5657–62. doi: 10.1073/pnas.96.10.5657 PMC21916 10318940

[42] Klein RS Garber C Howard N. Infectious immunity in the central nervous system and brain function. Nat Immunol (2017) 18:132–41. doi: 10.1038/ni.3656 PMC5815515 28092376

[43] Lindemann L Jaeschke G Michalon A Vieira E Honer M Spooren W. CTEP: a novel, potent, long-acting, and orally bioavailable metabotropic glutamate receptor 5 inhibitor. J Pharmacol Exp Ther (2011) 339:474–86. doi: 10.1124/jpet.111.185660 21849627

[44] Escartin C Galea E Lakatos A O'Callaghan JP Petzold GC Serrano-Pozo A. Reactive astrocyte nomenclature, definitions, and future directions. Nat Neurosci (2021) 24:312–25. doi: 10.1038/s41593-020-00783-4 PMC8007081 33589835

[45] Cardozo PL de Lima IBQ Maciel EMA Silva NC Dobransky T Ribeiro FM. Synaptic elimination in neurological disorders. Curr Neuropharmacol (2019) 17:1071–95. doi: 10.2174/1570159X17666190603170511 PMC7052824 31161981

[46] Kwart D Gregg A Scheckel C Murphy EA Paquet D Duffield M. A Large Panel of Isogenic APP and PSEN1 Mutant Human iPSC Neurons Reveals Shared Endosomal Abnormalities Mediated by APP beta-CTFs, Not Abeta. Neuron (2019) 104:256–270.e255. doi: 10.1016/j.neuron.2019.07.010 31416668

[47] Molla Kazemiha V Shokrgozar MA Arabestani MR Shojaei Moghadam M Azari S Maleki S. PCR-based detection and eradication of mycoplasmal infections from various mammalian cell lines: a local experience. Cytotechnology (2009) 61:117–24. doi: 10.1007/s10616-010-9252-6 PMC2825298 20135349

[48] Untergasser A Nijveen H Rao X Bisseling T Geurts R Leunissen JA. Primer3Plus, an enhanced web interface to Primer3. Nucleic Acids Res (2007) 35:71–4. doi: 10.1093/nar/gkm306 PMC1933133 17485472

[49] Kilkenny C Browne WJ Cuthill IC Emerson M Altman DG. Improving bioscience research reporting: the ARRIVE guidelines for reporting animal research. PloS Biol (2010) 8:e1000412. doi: 10.1371/journal.pbio.1000412 20613859 PMC2893951

[50] Villasana LE Klann E Tejada-Simon MV. Rapid isolation of synaptoneurosomes and postsynaptic densities from adult mouse hippocampus. J Neurosci Methods (2006) 158:30–6. doi: 10.1016/j.jneumeth.2006.05.008 PMC2014514 16797717

[51] Zamanian JL Xu L Foo LC Nouri N Zhou L Giffard RG. Genomic analysis of reactive astrogliosis. J Neurosci (2012) 32:6391–410. doi: 10.1523/JNEUROSCI.6221-11.2012 PMC3480225 22553043

[52] Liddelow SA Guttenplan KA Clarke LE Bennett FC Bohlen CJ Schirmer L. Neurotoxic reactive astrocytes are induced by activated microglia. Nature (2017) 541:481–7. doi: 10.1038/nature21029 PMC5404890 28099414

[53] Fan YY Huo J. A1/A2 astrocytes in central nervous system injuries and diseases: Angels or devils? Neurochem Int (2021) 148:105080. doi: 10.1016/j.neuint.2021.105080 34048845

[54] Mills EA Davis CH Bushong EA Boassa D Kim KY Ellisman MH. Astrocytes phagocytose focal dystrophies from shortening myelin segments in the optic nerve of Xenopus laevis at metamorphosis. Proc Natl Acad Sci U.S.A. (2015) 112:10509–14. doi: 10.1073/pnas.1506486112 PMC4547286 26240339

[55] Konishi H Koizumi S Kiyama H. Phagocytic astrocytes: Emerging from the shadows of microglia. Glia (2022) 70:1009–26. doi: 10.1002/glia.24145 PMC9305589 35142399

[56] Kim SK Hayashi H Ishikawa T Shibata K Shigetomi E Shinozaki Y. Cortical astrocytes rewire somatosensory cortical circuits for peripheral neuropathic pain. J Clin Invest (2016) 126:1983–97. doi: 10.1172/JCI82859 PMC4855913 27064281

[57] Danjo Y Shigetomi E Hirayama YJ Kobayashi K Ishikawa T Fukazawa Y. Transient astrocytic mGluR5 expression drives synaptic plasticity and subsequent chronic pain in mice. J Exp Med (2022) 219. doi: 10.1084/jem.20210989 PMC8952801 35319723

[58] de Souza JM Ferreira-Vieira TH Maciel EMA Silva NC Lima IBQ Doria JG. mGluR5 ablation leads to age-related synaptic plasticity impairments and does not improve Huntington's disease phenotype. Sci Rep (2022) 12:8982. doi: 10.1038/s41598-022-13029-z 35643779 PMC9148310

[59] Spurrier J Nicholson L Fang XT Stoner AJ Toyonaga T Holden D. Reversal of synapse loss in Alzheimer mouse models by targeting mGluR5 to prevent synaptic tagging by C1Q. Sci Transl Med (2022) 14:eabi8593. doi: 10.1126/scitranslmed.abi8593 35648810 PMC9554345

[60] Spampinato SF Copani A Nicoletti F Sortino MA Caraci F. Metabotropic glutamate receptors in glial cells: A new potential target for neuroprotection? Front Mol Neurosci (2018) 11:414. doi: 10.3389/fnmol.2018.00414 30483053 PMC6243036

[61] Casley CS Lakics V Lee HG Broad LM Day TA Cluett T. Up-regulation of astrocyte metabotropic glutamate receptor 5 by amyloid-beta peptide. Brain Res (2009) 1260:65–75. doi: 10.1016/j.brainres.2008.12.082 19401173

[62] Lim D Iyer A Ronco V Grolla AA Canonico PL Aronica E. Amyloid beta deregulates astroglial mGluR5-mediated calcium signaling *via* calcineurin and Nf-kB. Glia (2013) 61:1134–45. doi: 10.1002/glia.22502 23616440

[63] Grolla AA Fakhfouri G Balzaretti G Marcello E Gardoni F Canonico PL. Abeta leads to Ca(2)(+) signaling alterations and transcriptional changes in glial cells. Neurobiol Aging (2013) 34:511–22. doi: 10.1016/j.neurobiolaging.2012.05.005 22673114

[64] Grolla AA Sim JA Lim D Rodriguez JJ Genazzani AA Verkhratsky A. Amyloid-beta and Alzheimer's disease type pathology differentially affects the calcium signalling toolkit in astrocytes from different brain regions. Cell Death Dis (2013) 4:e623. doi: 10.1038/cddis.2013.145 23661001 PMC3674354

[65] Ronco V Grolla AA Glasnov TN Canonico PL Verkhratsky A Genazzani AA. Differential deregulation of astrocytic calcium signalling by amyloid-beta, TNFalpha, IL-1beta and LPS. Cell Calcium (2014) 55:219–29. doi: 10.1016/j.ceca.2014.02.016 24656753

[66] Mao L Wang JQ. Phosphorylation of cAMP response element-binding protein in cultured striatal neurons by metabotropic glutamate receptor subtype 5. J Neurochem (2003) 84:233–43. doi: 10.1046/j.1471-4159.2003.01256.x 12558986

[67] Wang H Zhuo M. Group I metabotropic glutamate receptor-mediated gene transcription and implications for synaptic plasticity and diseases. Front Pharmacol (2012) 3:189. doi: 10.3389/fphar.2012.00189 23125836 PMC3485740

[68] Doria JG de Souza JM Andrade JN Rodrigues HA Guimaraes IM Carvalho TG. The mGluR5 positive allosteric modulator, CDPPB, ameliorates pathology and phenotypic signs of a mouse model of Huntington's disease. Neurobiol Dis (2015) 73:163–73. doi: 10.1016/j.nbd.2014.08.021 25160573

[69] Doria JG de Souza JM Silva FR Olmo IG Carvalho TG Alves-Silva J. The mGluR5 positive allosteric modulator VU0409551 improves synaptic plasticity and memory of a mouse model of Huntington's disease. J Neurochem (2018) 147:222–39. doi: 10.1111/jnc.14555 PMC6317718 30028018

[70] Lee JH Kim JY Noh S Lee H Lee SY Mun JY. Astrocytes phagocytose adult hippocampal synapses for circuit homeostasis. Nature (2021) 590:612–7. doi: 10.1038/s41586-020-03060-3 33361813

[71] Gomez-Arboledas A Davila JC Sanchez-Mejias E Navarro V Nunez-Diaz C Sanchez-Varo R. Phagocytic clearance of presynaptic dystrophies by reactive astrocytes in Alzheimer's disease. Glia (2018) 66:637–53. doi: 10.1002/glia.23270 PMC5814816 29178139

[72] Iram T Trudler D Kain D Kanner S Galron R Vassar R. Astrocytes from old Alzheimer's disease mice are impaired in Abeta uptake and in neuroprotection. Neurobiol Dis (2016) 96:84–94. doi: 10.1016/j.nbd.2016.08.001 27544484

[73] Sanchez-Mico MV Jimenez S Gomez-Arboledas A Munoz-Castro C Romero-Molina C Navarro V. Amyloid-beta impairs the phagocytosis of dystrophic synapses by astrocytes in Alzheimer's disease. Glia (2021) 69:997–1011. doi: 10.1002/glia.23943 33283891

[74] Krabbe G Halle A Matyash V Rinnenthal JL Eom GD Bernhardt U. Functional impairment of microglia coincides with Beta-amyloid deposition in mice with Alzheimer-like pathology. PloS One (2013) 8:e60921. doi: 10.1371/journal.pone.0060921 23577177 PMC3620049

