# Supplementary material for: Blockade of mGluR5 in astrocytes derived from human iPSCs modulates astrocytic function and increases phagocytosis
Source: Front Immunol. 2023 Dec 11;14:1283331. doi: 10.3389/fimmu.2023.1283331 (PMC10749358; doi:10.3389/fimmu.2023.1283331)
Supplement: Supplementary file 1 [file DataSheet_1.pdf]

Supplementary Material

1 Supplementary Figures

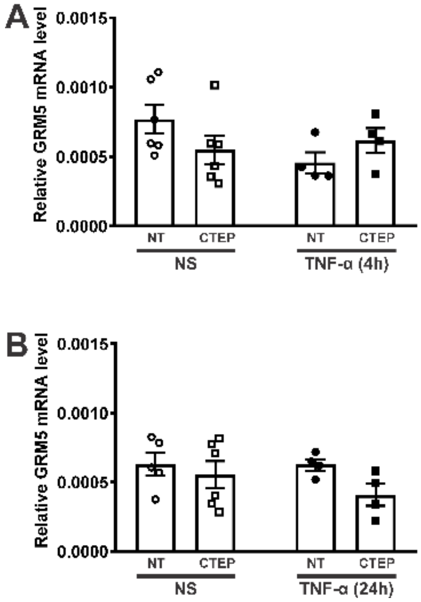

Figure S1: mGluR5 expression in hiPSC-derived astrocytes. Graphs show mRNA levels of mGluR5 (GRM5) in hiPSC-derived astrocytes that were either unstimulated (NS) or stimulated with rTNF-α 10 ng/mL and treated with either vehicle (NT) or CTEP 10 µM for either 4 h (A) or 24 h (B). mRNA levels were assessed by quantitative RT-PCR, which was performed in triplicates and normalized to the average of *RPLP0* and *IPO8* mRNA levels. Data represents the means ± SEM, n=4-6.

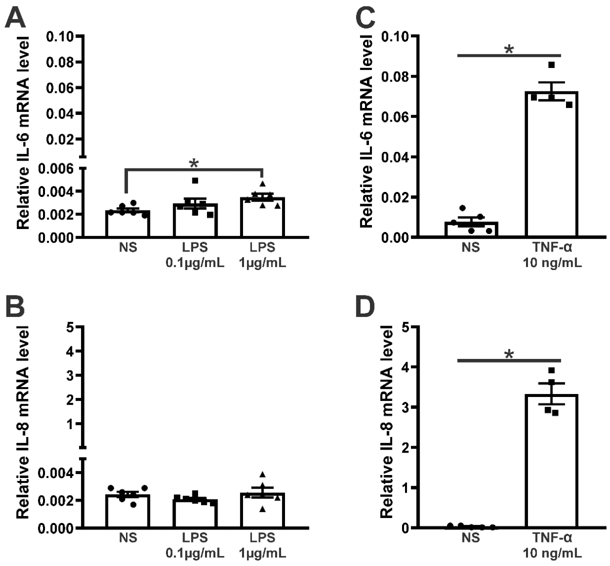

Figure S2: Expression of cytokines by hiPSC-derived astrocytes upon stimulation with LPS and rTNF-α. Graphs show mRNA levels of IL-6 (A) and IL-8 (B) in hiPSC-derived astrocytes that were either unstimulated (NS) or stimulated with LPS 0.1 or 1 μg/mL for 24 h. Graphs show mRNA levels of IL-6 (A) and IL-8 (B) in hiPSC-derived astrocytes that were either unstimulated (NS) or stimulated with rTNF-α 10 ng/mL for 24 h. mRNA levels were assessed by quantitative RT-PCR, which was performed in triplicates and normalized to the average of RPLP0 and IPO8 mRNA levels. Data represents the means ± SEM, N=4-6.

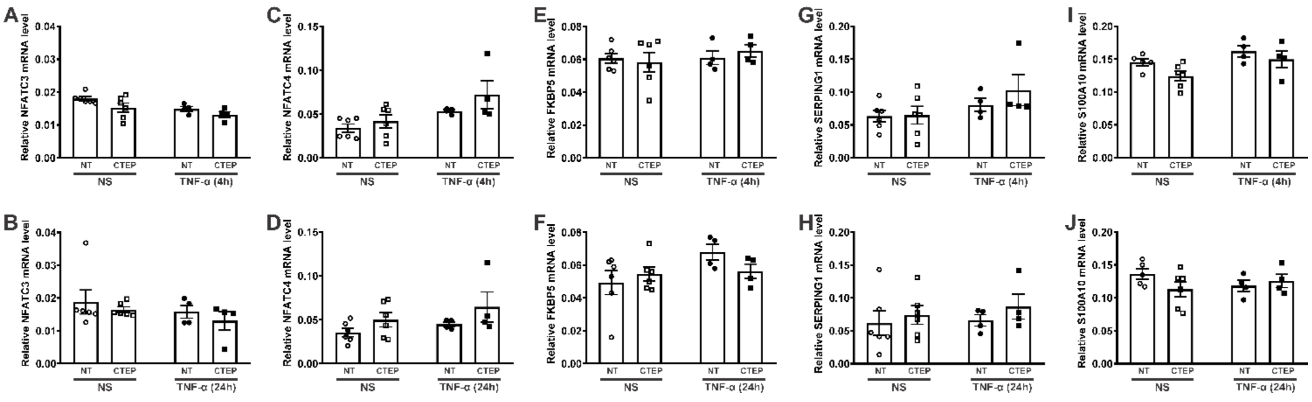

Figure S3: Expression of reactive astrocyte markers by hiPSC-derived astrocytes. Graphs show mRNA levels of NFATC3 (A), NFATC4 (C), FKBP5 (E), SERPING1 (G), and S100A10 (I) in hiPSC-derived astrocytes that were either unstimulated (NS) or stimulated with rTNF-α 10 ng/mL and treated with either vehicle (NT) or CTEP 10 μM for 4 h. Graphs show mRNA levels of NFATC3 (B), NFATC4 (D), FKBP5 (F), SERPING1 (H), and S100A10 (J) in hiPSC-derived astrocytes that were either unstimulated (NS) or stimulated with rTNF-α 10 ng/mL and treated with either vehicle (NT) or CTEP 10 μM for 24 h. mRNA levels were assessed by quantitative RT-PCR, which was performed in triplicates and normalized to the average of RPLP0 and IPO8 mRNA levels. Data represents the means ± SEM, N=4-6.

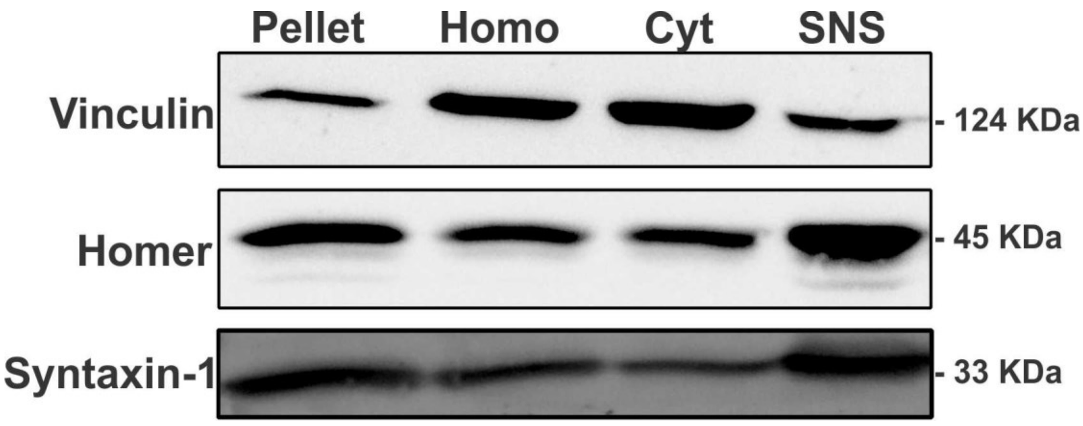

Figure S4: Synaptoneurosomes isolated from mouse brain are enriched in pre- and post-synaptic markers. Shown are immunoblots for vinculin (upper panel), Homer (middle panel) and syntaxin-1 (lower panel) expression in synaptoneurosomes preparation fractions, including pellet, homogenate (Homo), cytosolic (Cyt) and synaptoneurosomes (SNS).

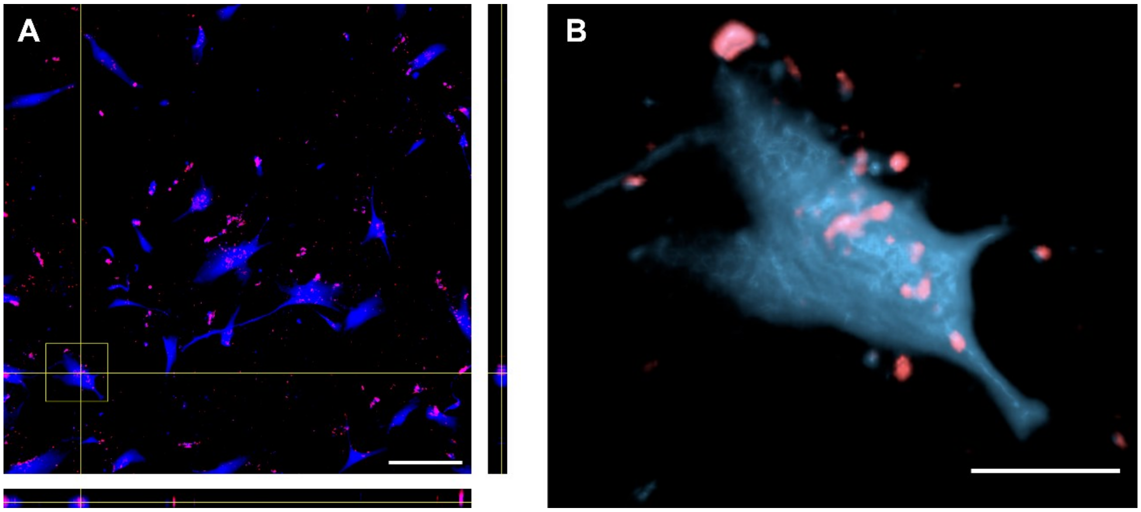

Figure S5: hiPSC-derived astrocytes phagocytose synaptoneurosomes. Shown are orthogonal projection of z-series (A) and 3D renderization (B) of astrocyte highlighted in (A) from confocal micrographs of hiPSC-derived astrocytes labelled with CellTracker blue and synpatoneurosomes labelled with Vybrant CM-Dil (red). Scale bar in (A)=200 µm and in (B)=50 µm.
